# Effect of Iron Therapy on Platelet Counts in Patients with Inflammatory Bowel Disease-Associated Anemia

**DOI:** 10.1371/journal.pone.0034520

**Published:** 2012-04-10

**Authors:** Stefanie Kulnigg-Dabsch, Rayko Evstatiev, Clemens Dejaco, Christoph Gasche

**Affiliations:** 1 Department of Medicine 3, Medical University of Vienna, Vienna, Austria; 2 Christian Doppler Laboratory for Molecular Carcinoma Chemoprevention, Medical University of Vienna, Vienna, Austria; University of Montreal, Canada

## Abstract

**Background and Aims:**

Secondary thrombocytosis is a clinical feature of unknown significance. In inflammatory bowel disease (IBD), thrombocytosis is considered a marker of active disease; however, iron deficiency itself may trigger platelet generation. In this study we tested the effect of iron therapy on platelet counts in patients with IBD-associated anemia.

**Methods:**

Platelet counts were analyzed before and after iron therapy from four prospective clinical trials. Further, changes in hemoglobin, transferrin saturation, ferritin, C-reactive protein, and leukocyte counts, before and after iron therapy were compared. In a subgroup the effect of erythropoietin treatment was tested. The results were confirmed in a large independent cohort (FERGIcor).

**Results:**

A total of 308 patient records were available for the initial analysis. A dose-depended drop in platelet counts (mean 425 G/L to 320 G/L; p<0.001) was found regardless of the type of iron preparation (iron sulphate, iron sucrose, or ferric carboxymaltose). Concomitant erythropoietin therapy as well as parameters of inflammation (leukocyte counts, C-reactive protein) had no effect on the change in platelet counts. This effect of iron therapy on platelets was confirmed in the FERGIcor study cohort (n=448, mean platelet counts before iron therapy: 383 G/L, after: 310 G/L, p<0.001).

**Conclusion:**

Iron therapy normalizes elevated platelet counts in patients with IBD-associated anemia. Thus, iron deficiency is an important pathogenetic mechanism of secondary thrombocytosis in IBD.

## Introduction

Platelets (thrombocytes) are small anuclear cell fragments that derive from mature megakaryocytes. Adult humans produce about 100 billion thrombocytes per day. The primary role of thrombocytes in mammalians is to ensure hemostasis by binding to von-Willebrand factor and fibrinogen. In addition, platelets are a source of pro-inflammatory and anti-microbial mediators [1]–[3]. An increase in the circulating number of platelets, i.e. thrombocytosis, may occur under certain circumstances such as neoplastic proliferative diseases (i.e. essential thrombocytosis) or secondary to other conditions such as hypo- or asplenism, but is also found with acute and chronic inflammation, malignant disease, blood loss or iron deficiency [4]. Except for hypo- or asplenism, the mechanism underlying secondary thrombocytosis and its clinical significance are not completely understood. Enhanced megakaryopoiesis may result from an increase of megakaryocytic growth factors such as thrombopoietin, interleukin (IL)-3, IL-6 or IL-11 [5]. However, the data on this are vague and further investigation of this topic is needed.

About one third of patients with inflammatory bowel disease (IBD) suffer from anemia [6]–[9]. IBD-associated anemia is caused by a combination of anemia of chronic disease and iron deficiency [10]. Accordingly, guidelines recommend iron replacement in combination with anti-inflammatory drugs and/or erythropoietin [11], [12]. Secondary thrombocytosis is another clinical feature of IBD that has been associated with active disease [13]. Some authors propose that activated platelets are involved in the pathogenesis of IBD [14], [15]. So far the relative importance of iron deficiency for IBD-associated thrombocytosis has not been elucidated. Here we tested the effect of iron therapy on changes in platelet counts in two large cohorts of patients with IBD-associated anemia.

## Results

**Figure 1:**
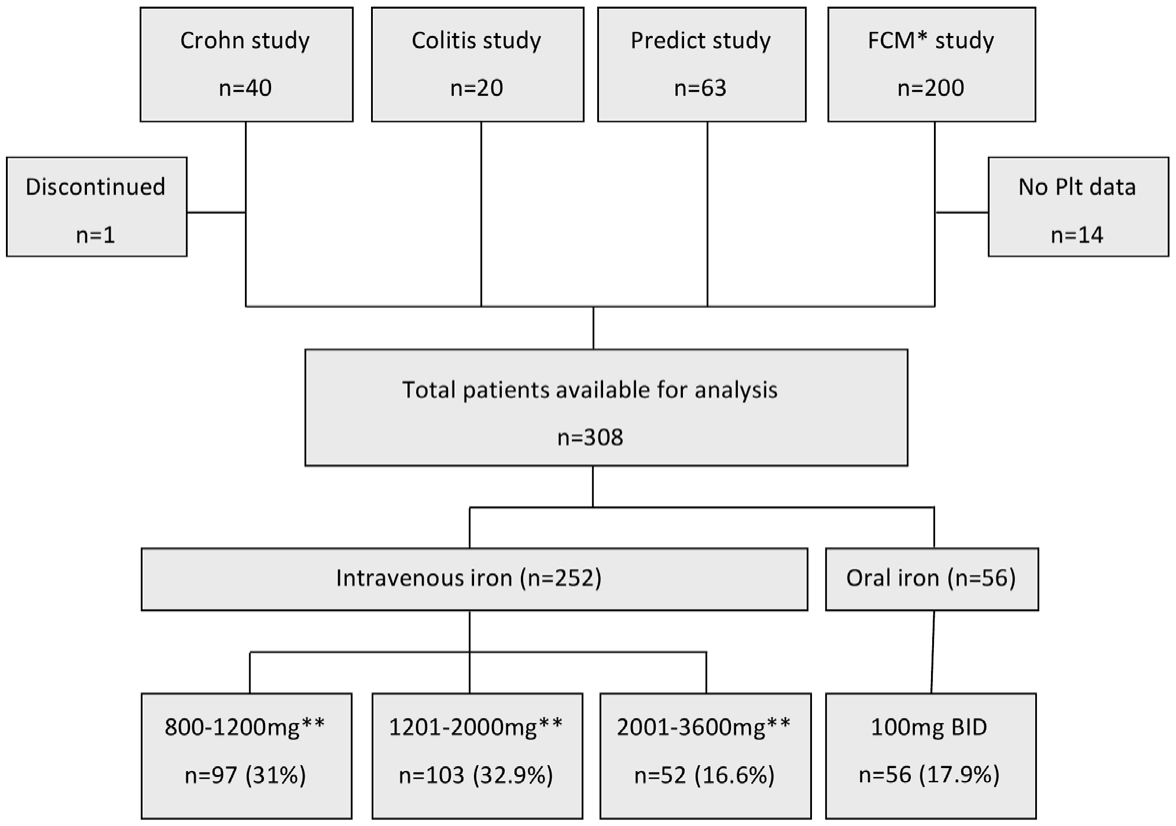
Primary dataset analysis. * FCM=ferric carboxymaltose. ** cumulative iron dose. Plt=platelets.

**Figure 2:**
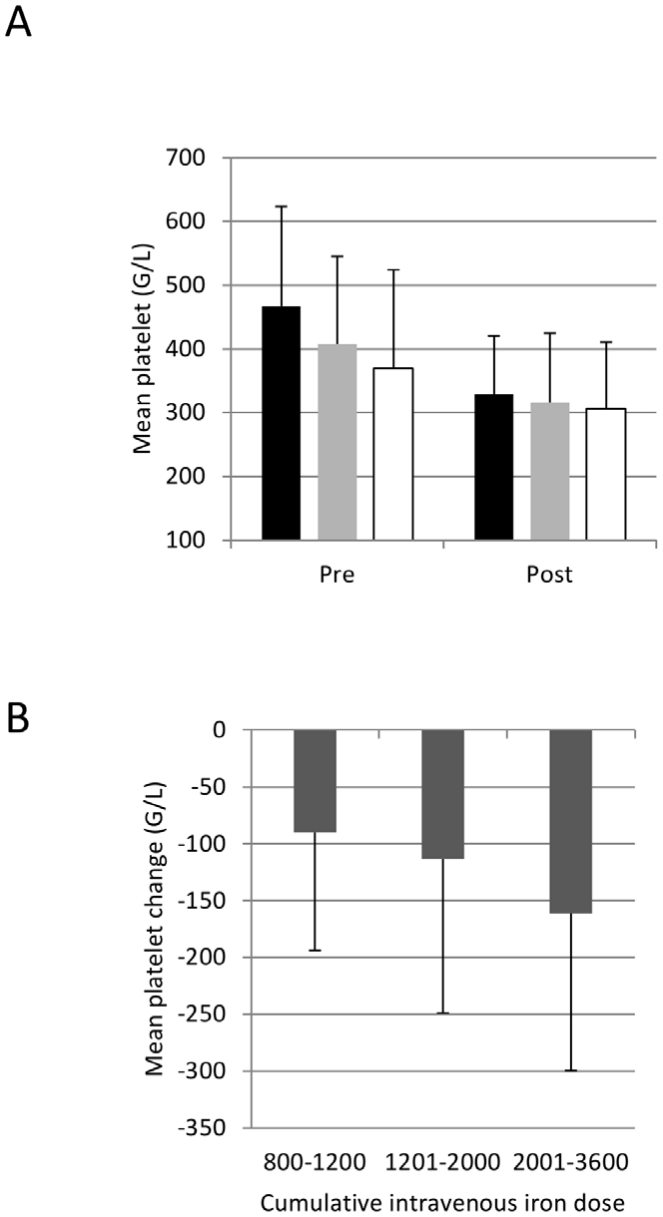
The changes in platelet counts upon iron therapy. (A) Platelet counts pre and post iron replacement therapy according to iron preparation (black column – iron sucrose n=122, grey column – ferric carboxymaltose n=130, white column – iron sulphate n=56), p<0.001 pre vs. post for all iron preparations. (B) Mean drop in platelet counts upon intravenous iron replacement therapy according to the cumulative iron dose (p=0.002). Group 0–1200 mg n=97, group 1201–2000 mg n=103, group 2001–3600 mg n=52. Error bars represent standard deviation.

The initial cohort consisted of a total of 323 patients (40 from the Crohn study, 20 from the Colitis Study, 63 from the Predict Study, and 200 from the Ferric carboxymaltose Study, Figure 1). From 308 patients platelets data were available for analysis. Patients were further divided into “intravenous iron" (three dose groups: “800–1200 mg iron", “1201–2000 mg iron", “ 2001–3600 mg iron") and “oral iron" (200 mg per day). Overall, the mean (SD) platelet counts dropped from 425 (153) G/L at study entry to 320 (101) G/L after iron treatment (table 1, p<0.001). This effect was observed with both intravenous (iron sucrose or ferric carboxymaltose) and oral (iron sulphate) iron products (figure 2A) and was dose-dependent (figure 2B, p=0.002). Hemoglobin, transferrin saturation and ferritin improved as expected, C-reactive protein and leukocyte counts did not change (table 1).

**Table 1:** Changes in laboratory parameters upon iron treatment (primary dataset n=307).

	Pre iron	Post iron	N	p value
Platelets G/L	425 (153)	320 (101)	308	<0.001
Hemoglobin g/dL	8.8 (1.4)	12.2 (1.7)	308	<0.001
Leukocytes G/L	8.9 (3.3)	8.7 (3.2)	121	0.577
C-reactive protein mg/dL*	0.8 (0–15.2)	0.7 (0–24)	303	0.735
Ferritin µg/L*	6 (0–407)	68 (1–1920)	301	<0.001
Transferrin saturation %*	3 (0.5–49)	16.1 (1–98)	302	<0.001

Continuous data given as mean (SD) or *median (range).

**Figure 3:**
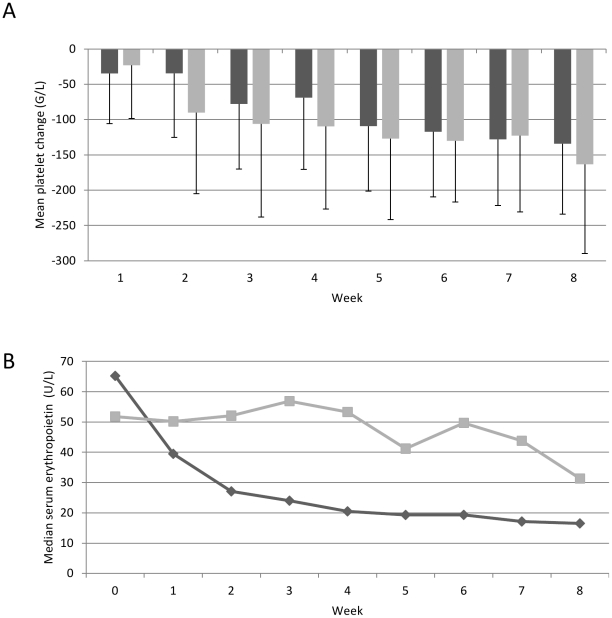
The changes of platelet counts and erythropoietin levels. Continuous drop in platelet counts (A, p=0.703) and serum erythropoietin levels (B, p=0.021) in Crohn's patients receiving iron sucrose (200–400 mg/week) and erythropoietin (150 mg/KG 3 times per week; light column/line) or iron sucrose (200–400 mg/week) and placebo (dark column/line). N=20 for each group. Error bars represent standard deviation.

In the “Crohn study" patients had been randomized to erythropoietin or placebo treatment but all patients had received iron sucrose. To test whether erythropoietin may interfere with the drop in platelet counts, the two treatment groups were compared and serum erythropoietin levels were analyzed every week. Platelets dropped to a similar extent and speed in the erythropoietin and the placebo group (average change in platelet counts per week −3.7% [95% CI −2.5, −4.9] versus −4.0% [−3.0, −5.0]; p=0.703) despite different changes of serum erythropoietin levels (average change in erythropoietin levels per week −5.5% [95% CI 0.4, −11] versus −14% [−9.8, −18] p=0.021, figure 3). These data suggest that erythropoietin had no effect on the change in platelet levels upon iron therapy.

**Figure 4:**
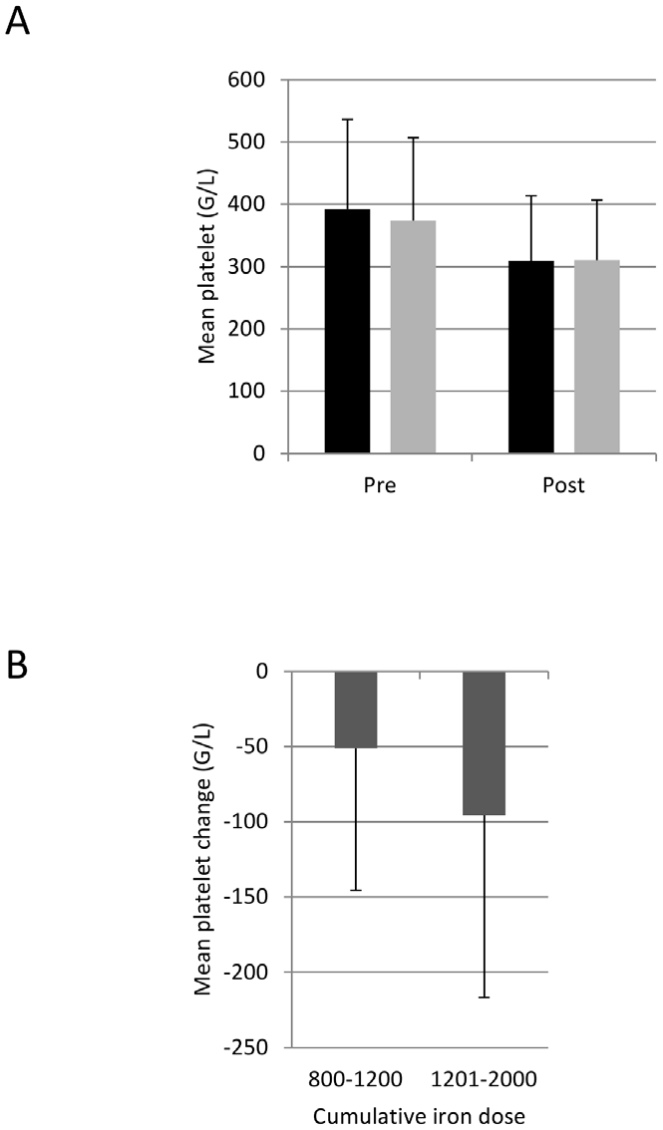
The changes in platelet counts in the FERGIcor trial. (A) Platelet counts pre and post iron therapy according to the iron preparation (black column – ferric carboxymaltose n=228, grey column – iron sucrose n=220), p<0.001 pre vs. post for both iron preparations. (B) Mean drop in platelets upon iron therapy according to the cumulative iron dose (p<0.001). Group 800–1200 mg n=228, group 1201–2000 mg n=220. Error bars represent standard deviation.

To confirm these unexpected findings we also analyzed a recent independent cohort of patients (from FERGIcor; [16]). From the total of 485 patients, platelet data were available from 448 patients pre and post iron therapy. 228 patients had received 500–1200 mg intravenous iron (50.9%) and 220 had received 1201–2000 mg (49.1%). Half of the patients were treated with ferric carboxymaltose (n=228, 50.9%), half with iron sucrose (n=220, 49.1%). In this cohort, the platelet counts dropped from mean 383 (SD 139) G/L at study entry to 310 (101) G/L after treatment (table 2, p<0.001) confirming the results from the initial analysis. The change in platelet counts was independent of the iron compound (figure 4A) and was dose-dependent (p<0.001, figure 4B). Similar to the initial cohort, hemoglobin and iron parameters improved but inflammation parameters did not change upon iron treatment (table 2).

**Table 2:** Changes in laboratory parameters upon iron treatment (confirmation dataset: FERGIcor, n=448).

	Pre iron	Post iron	N	p value
Platelets G/L	383 (139)	310 (101)	448	<0.001
Hemoglobin g/dL	10.2 (1.5)	12.8 (1.5)	427	<0.001
Leukocytes G/L	7.2 (2.6)	7.0 (2.5)	400	0.142
C-reactive protein mg/dL*	0.4 (0–6.0)	0.4 (0–5.8)	366	0.052
Ferritin µg/L*	8 (2–151)	72 (2–544)	420	<0.001
Transferrin saturation %*	5.6 (1.4–50)	19.1 (1.7–58)	402	<0.001

Continuous data given as mean (SD) or *median (range).

## Discussion

The relative importance of iron deficiency for IBD-associated anemia has been demonstrated in several studies. Here we addressed the hypothesis that iron deficiency is also relevant for IBD-associated thrombocytosis, a frequent finding in active IBD. In both cohorts iron therapy was associated with a dose-dependent normalization of elevated platelet counts. The decrease in platelets was observed upon oral iron sulphate as well as intravenous iron sucrose or ferric carboxymaltose treatment, and was independent of erythropoietin. As inflammation parameters were low and did not change during iron treatment we may conclude that IBD-associated thrombocytosis in our cohorts is a result of iron deficiency rather than systemic inflammation.

So far, the origin, the mechanism and consequences of thrombocytosis in IBD have been unknown. This study is the first to underline the relevance of iron deficiency in this setting. It is important to state that iron therapy did not lower platelet counts below physiological levels, indicating a regulatory rather than a toxic effect. Also the effect was seen with different iron preparations, which excludes a potential effect of the sugar carrier molecule (i.e. sucrose, carboxymaltose). As platelet counts decreased continuously, we surmise that iron therapy reduced the dynamics of megakaryopoiesis rather than changed the platelet half-life. Interestingly, concomitant erythropoietin treatment did not alter platelet dynamics though erythropoietin has been considered to stimulate megakaryopoiesis [17]. In vitro, erythropoietin has no effect on megakaryocytes and previous in vivo observations in patients with chronic renal failure were also considered a consequence of iron deficiency [18], [19]. We may speculate that iron deficiency has a direct effect on megakaryopoiesis to stimulate platelet production for counteracting chronic bleeding.

Iron deficiency is one of the world's most common and potentially treatable health problems, nevertheless little is known about its association with thrombocytosis. A Medline search performed in February 2012 with “thrombocytosis" and “iron deficiency" as search terms (limit English-language) identified 88 publications, mainly case reports. The first observations were reported in 1904 [20] when the effect of severe hemorrhage on the number of platelets was tested in rabbits. Several case reports linked thrombosis, thrombocytosis and iron deficiency [21]–[27]. More recently, this association was also discussed in patients with renal failure [19], [28]. However, this is the first study to demonstrate an effect of iron therapy on platelets counts in humans. A second Medline search performed in July 2011 with “thrombocytosis" and “IBD" as search terms identified 65 publications (limit English-language). Again, several case reports about thrombosis or thrombocytosis and IBD were identified [29]–[35]. Three studies [36]–[38] showed a correlation with high disease activity, one study in children found thrombocytosis only in endoscopically confirmed severe colitis [39]. In the study of Okun et al [40] the preoperative thrombocytosis was a risk factor for postoperative chronic pouchitis, however, in the study of Lian et al [41] it was not. Higher levels of thrombopoietin were found [42] in patients with active disease compared to inactive disease or healthy controls, however, thrombopoietin levels did not correlate with platelet levels. Thrombocytosis was shown to be a risk factor for steroid-dependency in IBD [43] or lack of response to cyclosporine [44]. None of these studies evaluated iron parameters in the context of thrombocytosis again underlining the originality of our study.

As to the retrospective nature of this study certain shortcomings have to be considered. It is necessary to note that all patients had anemia and IBD, a diagnostic combination that selected for the presence of iron deficiency. Inclusion criteria and treatment regimen (iron preparation and dosage) varied between studies which might have had an impact on the outcome. Another shortcoming is the absence of non-iron treatment control group. Unfortunately, none of the studies from which the databases were made available had such a control group. However, all iron compounds (both oral and intravenous) showed a similar effect indicating that it is a true iron rather than carrier effect. In addition in all studies IBD medications were restricted by the inclusion criteria and had to be stable before and during the respective clinical trials. When testing leukocyte counts and C-reactive protein no change was found. This indicates that the drop in platelets is rather not caused by changes in inflammation but by iron therapy itself. To strengthen our findings from the initial cohort and to better control for these shortcomings the data were confirmed in a second recently published cohort (FERGIcor) from 88 centers in 14 European countries. In this second cohort the absolute platelet counts before iron treatment were not as high as in the initial cohort; however, anemia and, accordingly, iron deficiency were also not as severe. Post iron therapy platelet counts in both cohorts were comparable.

In summary, iron therapy normalizes elevated platelet counts in patients with IBD-associated anemia. Thus, our study provides initial evidence for a direct association between iron deficiency and secondary thrombocytosis in IBD-associated anemia. In cancer patients thrombocytosis is an independent risk factor for thromboembolic events [45]. In IBD, predictive laboratory parameters for thromboembolic events have not been evaluated. It will be important to assess platelet activity and clot formation in this setting. Further the role of secondary thrombocytosis in IBD patients without iron deficiency needs clarification. It is tempting to speculate that thrombocytosis in chronic disease arises on the basis of an iron-deficient megakaryopoiesis, when iron is kept in the monocyte-macrophage-system and thus is unavailable to the bone marrow. Interventional studies in non-anemic IBD and cancer patients are under way. If our hypothesis is true and iron deficiency is causatively linked to thromboembolic events our work has strong impact on medical practice in various fields including orthopedic surgery, cancer, and chronic disease.

## Methods

### Study cohorts

The primary analysis was conducted at the Medical University of Vienna between June and September 2008. The local ethics committee had approved the protocol for the retrospective analysis (ethic committee of the Medical University of Vienna). Laboratory parameters before and after iron therapy (platelets, hemoglobin, leukocytes, C-reactive protein, ferritin, transferrin saturation) were obtained from four prospective clinical trials that had tested iron therapy with and without erythropoietin in IBD:

1. In the “Crohn study"[46], 40 patients with anemia and Crohn's disease receiving total dosages of up to 3600 mg iron sucrose over 16 weeks were randomized to erythropoietin treatment or placebo. Erythropoietin levels were measured in serum samples (stored at −20°C) using a commercial radioimmunoassay (Bio-Merieux, Nörtingen, Germany).
2. In the “Colitis study" [47], 20 patients with anemia and ulcerative colitis were treated with a total amount of 2000 mg iron sucrose over eight weeks. Non-responders to iron received additionally erythropoietin.
3. In the “Predict study" [48], 63 patients with anemia and IBD were treated with a total amount of 1200 mg iron sucrose over four weeks.
4. In the “Ferric carboxymaltose study" [49], 200 patients with anemia and IBD were randomized 2∶1 to ferric carboxymaltose (total median amount 1400 mg iron, n=137) or oral iron sulphate (200 mg/day, n=63). The endpoints were evaluated at week 12.

A second database from a recently published multicenter clinical trial (FERGIcor; [16]) was made available in October 2011 and used as confirmation cohort. This trial was conducted between October 2008 and December 2009. In the “FERGIcor study", 485 patients with IBD-associated anemia were randomized 1∶1 to ferric carboxymaltose (total mean amount 1380 mg iron, n=244) or iron sucrose (total mean amount 1160 mg iron, n=241). The endpoints were tested after 12 weeks. The study was registered at: ClinicalTrials.gov (NCT00810030).

All five prospective studies were approved by the local ethic committees as stated in the publications [16], [46]–[49], for the prospective studies written informed consent was obtained before the screening visit.

### Statistical analysis

Data were analyzed for distribution. Paired sample t-test or Wilcoxon test for paired samples were used to compare values before and after iron therapy as appropriate. Changes in platelet counts according to iron dose were analyzed by analysis of variance. Comparison of erythropoietin levels and platelet counts over time was performed after a log-transformation because of skewed distribution. The repeated weekly measurements were summarized per patient by computing the slopes from individual patient's linear regression analyses. The slopes were compared between groups by an independent samples t-test.

## References

[1] Linden MD, Jackson DE (2010). Platelets: Pleiotropic roles in atherogenesis and atherothrombosis.. Int J Biochem Cell Biol.

[2] McMorran BJ, Marshall VM, de Graaf GC, Drysdale KE, Shabbar M (2009). Platelets kill intraerythrocytic malarial parasites and mediate survival to infection.. Science.

[3] Danese S, Sans M, Fiocchi C (2004). The CD40/CD40L costimulatory pathway in inflammatory bowel disease.. Gut.

[4] Schafer AI (2001). Thrombocytosis and thrombocythemia.. Blood Rev.

[5] Ceresa IF, Noris P, Ambaglio C, Pecci A, Balduini CL (2007). Thrombopoietin is not uniquely responsible for thrombocytosis in inflammatory disorders.. Platelets.

[6] Gasche C (2000). Anemia in IBD: the overlooked villain.. Inflamm Bowel Dis.

[7] Stein J, Hartmann F, Dignass AU (2010). Diagnosis and management of iron deficiency anemia in patients with IBD.. Nat Rev Gastroenterol Hepatol.

[8] Gomollon F, Gisbert JP (2009). Anemia and inflammatory bowel diseases.. World J Gastroenterol.

[9] Kulnigg S, Gasche C (2006). Systematic review: managing anaemia in Crohn's disease.. Aliment Pharmacol Ther.

[10] Gasche C, Lomer MC, Cavill I, Weiss G (2004). Iron, anaemia, and inflammatory bowel diseases.. Gut.

[11] Gasche C, Berstad A, Befrits R, Beglinger C, Dignass A (2007). Guidelines on the diagnosis and management of iron deficiency and anemia in inflammatory bowel diseases.. Inflamm Bowel Dis.

[12] Gasche C, Evstatiev R, Haas T, Kaser A, Knoflach P (2011). [Diagnosis and treatment of iron deficiency and anaemia in inflammatory bowel diseases. Consensus of the Austrian IBD Working Party].. Z Gastroenterol.

[13] Harries AD, Fitzsimons E, Fifield R, Dew MJ, Rhoades J (1983). Platelet count: a simple measure of activity in Crohn's disease.. Br Med J (Clin Res Ed).

[14] Danese S, Motte Cd CL, Fiocchi C (2004). Platelets in inflammatory bowel disease: clinical, pathogenic, and therapeutic implications.. Am J Gastroenterol.

[15] Danese S, Scaldaferri F, Papa A, Pola R, Sans M (2004). Platelets: new players in the mucosal scenario of inflammatory bowel disease.. Eur Rev Med Pharmacol Sci.

[16] Evstatiev R, Marteau P, Iqbal T, Khalif IL, Stein J (2011). FERGIcor, a randomized controlled trial on ferric carboxymaltose for iron deficiency anemia in inflammatory bowel disease.. Gastroenterology.

[17] Beguin Y (1999). Erythropoietin and platelet production.. Haematologica.

[18] Loo M, Beguin Y (1999). The effect of recombinant human erythropoietin on platelet counts is strongly modulated by the adequacy of iron supply.. Blood.

[19] Streja E, Kovesdy CP, Greenland S, Kopple JD, McAllister CJ (2008). Erythropoietin, iron depletion, and relative thrombocytosis: a possible explanation for hemoglobin-survival paradox in hemodialysis.. Am J Kidney Dis.

[20] Richardson FL (1904). Effect of severe hemorrhage on the Number of Blood Plates in Blood from the Peripheral Circulation of Rabbits.. J Med Res.

[21] Basak R, Chowdhury A, Fatmi L, Saha N, Mollah A (2008). Stroke in the young: relationship with iron deficiency anemia and thrombocytosis.. Mymensingh Med J.

[22] Houissa F, Salem M, Bouzaidi S, Rejeb MB, Mekki H (2011). Cerebral thrombosis in inflammatory bowel disease: A report of four cases.. J Crohns Colitis.

[23] Benedict SL, Bonkowsky JL, Thompson JA, Van Orman CB, Boyer RS (2004). Cerebral sinovenous thrombosis in children: another reason to treat iron deficiency anemia.. J Child Neurol.

[24] Franchini M, Targher G, Montagnana M, Lippi G (2008). Iron and thrombosis.. Ann Hematol.

[25] Huang PH, Su JJ, Lin PH (2010). Iron deficiency anemia - a rare etiology of sinus thrombosis in adults.. Acta Neurol Taiwan.

[26] Keung YK, Owen J (2004). Iron deficiency and thrombosis: literature review.. Clin Appl Thromb Hemost.

[27] Kinoshita Y, Taniura S, Shishido H, Nojima T, Kamitani H (2006). Cerebral venous sinus thrombosis associated with iron deficiency: two case reports.. Neurol Med Chir (Tokyo).

[28] Vaziri ND (2009). Thrombocytosis in EPO-treated dialysis patients may be mediated by EPO rather than iron deficiency.. Am J Kidney Dis.

[29] Capron JP, Remond A, Lebrec D, Delamarre J, Dupas JL (1979). Gastrointestinal bleeding due to chronic portal vein thrombosis in ulcerative colitis.. Dig Dis Sci.

[30] Junge U, Wienke J, Schuler A (2001). Acute Budd-Chiari syndrome, portal and splenic vein thrombosis in a patient with ulcerative colitis associated with antiphospholipid antibodies and protein C deficiency.. Z Gastroenterol.

[31] Mijnhout GS, Klinkenberg EC, Lycklama G, Linskens R, Meuwissen SG (2004). Sepsis and elevated liver enzymes in a patient with inflammatory bowel disease: think of portal vein thrombosis.. Dig Liver Dis.

[32] Musio F, Older SA, Jenkins T, Gregorie EM (1993). Case report: cerebral venous thrombosis as a manifestation of acute ulcerative colitis.. Am J Med Sci.

[33] Schneiderman JH, Sharpe JA, Sutton DM (1979). Cerebral and retinal vascular complications of inflammatory bowel disease.. Ann Neurol.

[34] Standridge S, de los RE (2008). Inflammatory bowel disease and cerebrovascular arterial and venous thromboembolic events in 4 pediatric patients: a case series and review of the literature.. J Child Neurol.

[35] Thachil J (2008). Extreme thrombocytosis–an unusual presentation of inflammatory bowel disease.. Intern Med.

[36] Heits F, Stahl M, Ludwig D, Stange EF, Jelkmann W (1999). Elevated serum thrombopoietin and interleukin-6 concentrations in thrombocytosis associated with inflammatory bowel disease.. J Interferon Cytokine Res.

[37] Sethy PK, Dutta U, Aggrawal AN, Das R, Gulati M (2003). Pulmonary and hematological alterations in idiopathic ulcerative colitis.. Indian J Gastroenterol.

[38] Larsen TB, Nielsen JN, Fredholm L, Lund ED, Brandslund I (2002). Platelets and anticoagulant capacity in patients with inflammatory bowel disease.. Pathophysiol Haemost Thromb.

[39] Holmquist L, Ahren C, Fallstrom SP (1989). Relationship between results of laboratory tests and inflammatory activity assessed by colonoscopy in children and adolescents with ulcerative colitis and Crohn's colitis.. J Pediatr Gastroenterol Nutr.

[40] Okon A, Dubinsky M, Vasiliauskas EA, Papadakis KA, Ippoliti A (2005). Elevated platelet count before ileal pouch-anal anastomosis for ulcerative colitis is associated with the development of chronic pouchitis.. Am Surg.

[41] Lian L, Fazio VW, Lavery IC, Hammel J, Remzi FH (2009). Evaluation of association between precolectomy thrombocytosis and the occurrence of inflammatory pouch disorders.. Dis Colon Rectum.

[42] Papa A, Danese S, Piccirillo N, Toriani-Terenzi C, Bartolozzi F (2003). Thrombopoietin serum levels in patients with inflammatory bowel disease with and without previous thromboembolic events.. Hepatogastroenterology.

[43] Chow DK, Sung JJ, Tsoi KK, Wong VW, Wu JC (2009). Predictors of corticosteroid-dependent and corticosteroid-refractory inflammatory bowel disease: analysis of a Chinese cohort study.. Aliment Pharmacol Ther.

[44] Huaman Rios JW, Casellas JF, Malagelada Benapres JR (2009). Predictive factors of poor response to intravenous cyclosporine in steroid-refractory ulcerative colitis.. Rev Esp Enferm Dig.

[45] Simanek R, Vormittag R, Ay C, Alguel G, Dunkler D (2010). High platelet count associated with venous thromboembolism in cancer patients: results from the Vienna Cancer and Thrombosis Study (CATS).. J Thromb Haemost.

[46] Gasche C, Dejaco C, Waldhoer T, Tillinger W, Reinisch W (1997). Intravenous iron and erythropoietin for anemia associated with Crohn disease. A randomized, controlled trial.. Ann Intern Med.

[47] Gasche C, Dejaco C, Reinisch W, Tillinger W, Waldhoer T (1999). Sequential treatment of anemia in ulcerative colitis with intravenous iron and erythropoietin.. Digestion.

[48] Gasche C, Waldhoer T, Feichtenschlager T, Male C, Mayer A (2001). Prediction of response to iron sucrose in inflammatory bowel disease-associated anemia.. Am J Gastroenterol.

[49] Kulnigg S, Stoinov S, Simanenkov V, Dudar LV, Karnafel W (2008). A novel intravenous iron formulation for treatment of anemia in inflammatory bowel disease: the ferric carboxymaltose (FERINJECT) randomized controlled trial.. Am J Gastroenterol.

